# Methylation quantitative trait locus analysis of osteoarthritis links epigenetics with genetic risk

**DOI:** 10.1093/hmg/ddv433

**Published:** 2015-10-13

**Authors:** Michael D. Rushton, Louise N. Reynard, David A. Young, Colin Shepherd, Guillaume Aubourg, Fiona Gee, Rebecca Darlay, David Deehan, Heather J. Cordell, John Loughlin

**Affiliations:** 1 Musculoskeletal Research Group, Institute of Cellular Medicineand,; 2 Institute of Genetic Medicine, International Centre for Life, Newcastle University, Newcastle upon Tyne NE1 3BZ, UK and; 3 Freeman Hospital, High Heaton, Newcastle upon Tyne NE7 7DN, UK

## Abstract

Osteoarthritis (OA) is a common, painful and debilitating disease of articulating joints resulting from the age-associated loss of cartilage. Well-powered genetic studies have identified a number of DNA polymorphisms that are associated with OA susceptibility. Like most complex trait loci, these OA loci are thought to influence disease susceptibility through the regulation of gene expression, so-called expression quantitative loci, or eQTLs. One mechanism through which eQTLs act is epigenetic, by modulating DNA methylation. In such cases, there are quantitative differences in DNA methylation between the two alleles of the causal polymorphism, with the association signal referred to as a methylation quantitative trait locus, or meQTL. In this study, we aimed to investigate whether the OA susceptibility loci identified to date are functioning as meQTLs by integrating genotype data with whole genome methylation data of cartilage DNA. We investigated potential genotype–methylation correlations within a 1.0–1.5 Mb region surrounding each of 16 OA-associated single-nucleotide polymorphisms (SNPs) in 99 cartilage samples and identified four that function as meQTLs. Three of these replicated in an additional cohort of up to 62 OA patients. These observations suggest that OA susceptibility loci regulate the level of DNA methylation in *cis* and provide a mechanistic explanation as to how these loci impact upon OA susceptibility, further increasing our understanding of the role of genetics and epigenetics in this common disease.

## Introduction

Osteoarthritis (OA) is characterized by the age-related gradual thinning and eventual focal loss of articular cartilage and as such is a painful disease that severely impacts on normal joint function (1,2). There are no disease-modifying pharmacological therapies for OA, with pain management and joint replacement the principal clinical treatments. As a result, the health economic burden of the disease is large and will increase with a progressively ageing population.

OA is polygenic and a number of candidate gene and genome-wide association scan (GWAS) studies have reported OA risk-conferring loci for knee, hip or hand OA that exceed or are close to the genome-wide significance threshold (3–14). While some of the signals encompass genes with prior known roles in joint biology, most do not. In addition, many of the signals demonstrate association only in the knee, or the hip, or the hand rather than across all tested skeletal sites. Furthermore, some signals are restricted to a particular sex, an example being the association signal marked by the intergenic single-nucleotide polymorphism (SNP) rs10948172, which is only relevant to OA in males (11). Stratification by joint site and sex has therefore proven critical in identifying OA association signals (3).

A recurring feature of the GWAS data derived for common polygenic traits is that the majority of association signals do not alter gene-coding sequences but rather are located within non-coding or intergenic regions. It is assumed therefore that the polymorphisms are within regulatory elements and influence the trait by altering gene expression. Such loci are referred to as expression quantitative trait loci, or eQTLs (15). Examples in OA are the association signals marked by SNPs rs143383, rs4730250 and rs225014, which show correlation with the expression of the genes *GDF5*, *HBP1* and *DIO2*, respectively (5,16–19). These studies support a growing consensus that OA susceptibility loci act through regulating gene expression.

It is becoming increasingly apparent that epigenetics also has a prominent role in OA pathogenesis. For example, candidate studies have demonstrated that several genes involved in cartilage homeostasis are differentially methylated in OA (20–22), while more genome-wide approaches using DNA methylation arrays have highlighted compelling epigenetic alterations associated with the disease (23–27).

The relationship between DNA sequence variants and DNA methylation has generated a large amount of interest in recent years, and it is now well established that SNPs can influence the level of methylation at proximal CpG sites. Such SNPs are referred to as methylation quantitative trait loci or meQTLs. The identification of meQTLs can help in the interpretation of non-coding genetic risk alleles, potentially identifying novel disease-associated candidate genes in which methylation acts as a mediator between genotype and phenotype. This approach has been successfully utilized in a number of conditions, including neurological disorders, cancer, rheumatoid arthritis and metabolic traits (28–32). meQTLs are common in the human genome, with methylation levels of 28.5% of CpGs associated with nearby SNPs in adipose tissue (32) while 10.7% of type II diabetes risk SNPs are meQTLs (33). A specific example is the adipose-specific meQTL located in an enhancer region upstream of the metabolic disease-associated gene *ADCY3*. Two CpG sites within this region correlate with the genotype of the body mass index SNP rs713586. Between these two CpG sites is a SNP in perfect (*r*^2^ = 1) linkage disequilibrium (LD) with rs713586 that overlaps a binding site for USF1, a transcription factor that regulates genes involved in lipid and glucose homeostasis (32). In OA, evidence of interplay between genetics and epigenetics has been reported for the functional SNP rs143383, with its effect on *GDF5* expression being modulated by DNA methylation (34,35), and for the *DIO2* SNP rs225014 (36).

In this study, we aimed to investigate all of the OA association signals reported to date to assess which, if any, are functioning as meQTLs. We focussed on cartilage, the tissue central to the OA disease process. Cartilage contains only a single cell type, the chondrocyte, which in the context of epigenetic studies is an advantage in that it limits the scope for confounding heterogeneity that can be encountered if studying the epigenetics of a multicellular tissue in which cell-specific effects are likely. To identify meQTLs, we combined genotyping data with genome-wide methylation data from the cartilage of 99 patients, 63 of whom had knee OA, 17 with hip OA and 19 that had a neck of femur (NOF) fracture and who are free of OA. In total, we assessed 16 SNPs representing 16 loci that have been reported to be associated with OA.

## Results

### Identification of OA meQTLs

Genome-wide methylation was measured using our previously published data (24) using the Illumina Infinium HumanMethylation450 BeadChip. This array, which captures over 450 000 CpGs, will henceforth be referred to as the 450k array. For each of the 16 loci, we covered at least the whole of the LD region; therefore, for 15 loci we spanned 1 Mb while for the rs10948172 locus we spanned 1.5 Mb. Table 1 lists the loci and the number of CpG probes from the 450k array that were assessed at each locus for genotype–methylation correlations. Initially, we assessed correlations in all samples combined—OA knee, OA hip and NOF. This analysis identified four SNPs that correlated with methylation (Benjamin–Hochberg *P* < 0.05): chromosome 3p21.1 (*GLT8D1*), SNP rs6976 and three CpGs; chromosome 6p21.1 (*SUPT3H*), SNP rs10948172 and four CpGs; chromosome 15q21.3 (*ALDH1A2*), SNP rs3204689 and one CpG; and chromosome 20q11 (*GDF5*), SNP rs143383 and one CpG (Table 2). Patient information and genotypes at the four SNPs are shown in Supplementary Material, Table S1. For each of these nine CpGs, we next compared the methylation levels without genotype stratification but stratified by OA knee, OA hip and NOF. This revealed that the degree of methylation was on average significantly lower in the NOF patients for the 3p21.1 (*GLT8D1*, rs6976) and 6p21.1 (*SUPT3H*, rs10948172) CpGs, but that there was no difference between OA knee and OA hip (data not shown). We therefore repeated the analysis for all 16 loci separating OA from NOF. This analysis of only OA samples identified the same four SNPs and no OA-specific or NOF-specific genotype–methylation correlations (Table 2). As mentioned previously, many OA genetic association signals were only discovered following stratification by joint and/or sex. When we repeated our analysis using such stratification, we did not identify any further genotype–methylation correlations. In summary, we identified four potential meQTLs, and these are summarized in Table 2 and discussed in more detail below.

**Table 1.** List of the 16 OA association signals studied

Locus	Association SNP	Major/minor allele	Position (hg19)	Chr.	Nearest gene	Proxy SNP	Stratum	References	Number of CpG probes from the 450k array
1	rs6976	C/T	52703844	3	*GLT8D1*	–	Knees and hips	11	431
2	rs12107036	G/A	191082854	3	*TP63*	–	Female knees	11	76
3	rs10948172	A/G	44885669	6	*SUPT3H*	–	Males	11	197
4	rs9350591	C/T	76298247	6	*FILIP1*	rs7756065	Hips	11	157
5	rs3815148	A/C	106938420	7	*COG5*	rs1548524	Knees	8	135
6	rs4836732	T/C	118306516	9	*ASTN2*	–	Female hips	11	57
7	rs10492367	G/T	27906237	12	*KLHDC5*	rs10843013	Hips	11	123
8	rs835487	A/G	103584897	12	*CHST11*	–	Hips	11	190
9	rs11842874	A/G	113694259	13	*MCF2L*	–	Knees and hips	10	655
10	rs225014	T/C	80669330	14	*DIO2*	–	Hips	7	32
11	rs945006	T/G	102029277	14	*DIO3*	–	Knees and hips	9	385
12	rs3204689	C/G	58246802	15	*ALDH1A2*	–	Hands	13	91
13	rs8044769	C/T	53839135	16	*FTO*	–	Females	11	92
14	rs12982744	C/G	2177193	19	*DOT1L*	–	Male hips	12	135
15	rs6094710	G/A	46095649	20	*NCOA3*	–	Hips	14	71
16	rs143383	T/C	34025983	20	*GDF5*	rs6087704	Knees and hips	4,5	270

Stratum highlights the joint and/or sex that the signal shows association with from the original genetic study. The number of CpG probes from the 450k array that is present within the 1 Mb region (1.5 Mb for rs10948172) surrounding the association SNP are shown. In the discovery analysis, 90 of the 99 patients had their genotypes at the 16 SNPs determined using data from the HumanOmniExpress array. For SNPs not present on the array, a proxy SNP that had the highest linkage disequilibrium (LD) with the association SNP and which was on the array was used to infer genotypes. This was necessary for four SNPs; for three of these (rs9350591, rs3815148 and rs10492367), the proxy SNP is in perfect LD (*r*^2^ = 1) with the association SNP while for rs143383, the proxy SNP has an *r*^2^ of 0.93.

**Table 2.** List of significant genotype-methylation associations identified

SNP	CpG probe	Chr.	Probe location (hg19)	SNP location (hg19)	Adj. *P*-value All samples	Adj. *P*-value OA
rs6976	cg18099408 cg15147215 cg18591801	3p21.1	52552593 52552868 52553433	52728804	3.19 × 10^−8^ 0.005 0.004	3.73 × 10^−6^ 0.04 0.04
rs10948172	cg13979708 cg19254793 cg20913747 cg18551225	6p21.1	44695318 44695348 44695427 44695536	44777691	9.83 × 10^−6^ 0.008 8.75 × 10^−13^ 1.70 × 10^−10^	6.2 × 10^−5^ 0.009 4.9 × 10^−12^ 1.12 × 10^−10^
rs3204689	cg12031962	15q21.3	58353849	58246802	1.03 × 10^−10^	2.0 × 10^−8^
rs143383	cg14752227	20q11	34000481	34025983	0.001	0.01

Shown are the *P*-values from all of the samples combined and OA samples only. Adj. *P*-value represents the Benjamini–Hochberg corrected *P*-value.

**Figure 1.**
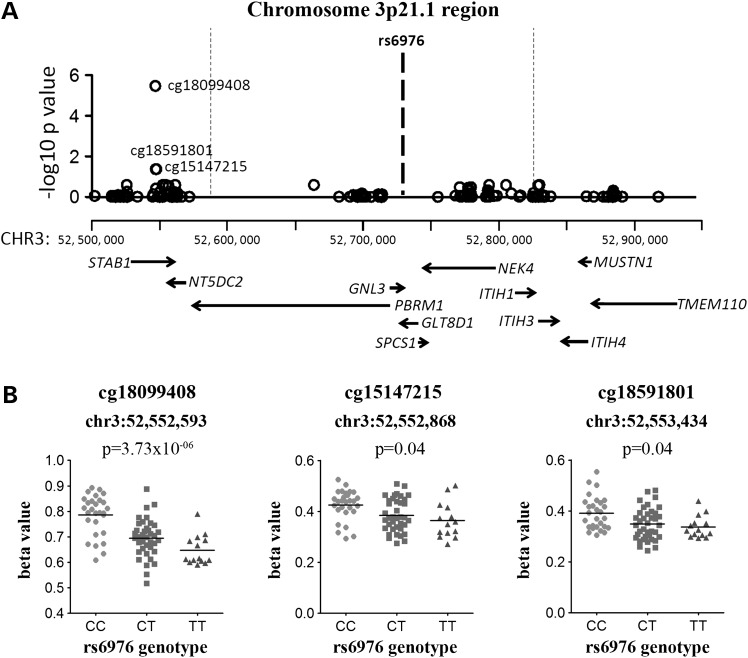
Genotype at rs6976 correlates with the methylation levels at three CpG sites within the gene body of *STAB1*. (**A**) The plot shows the association between rs6976 and the methylation levels of CpG probes that are present within the LD block. The *x*-axis represents the genomic position of the CpG probes, and the *y*-axis represents the Benjamini–Hochberg corrected −log_10_ *P*-value of the correlation between rs6976 genotype and β-value at each CpG probe. Each open circle represents a single CpG probe, and the three significant associations are labelled (cg15147215 and cg18591801 have the same *P*-value and on the *x*-axis scale used are represented by a single open circle). The LD block is indicated by the vertical dotted lines and the location of rs6976 by the bold dashed line. The genes within the region analysed are indicated below the association plot, with the gene direction indicated by arrows. (**B**) The association between genotype at rs6976 and methylation levels at the three significant CpG probes. Due to the significant methylation differences at these CpGs between OA and NOF samples, data are shown only for the 80 OA samples from the discovery cohort of 99 samples. The level of methylation at the CpG probes is shown as the β-value. Horizontal line represents the mean.

Genotype at *GLT8D1* SNP rs6976 correlated with the methylation of three CpGs located ∼31.5 kb upstream of the LD block and ∼170 kb from the SNP: cg18099408, cg15147215 and cg18591801. All three CpGs are within the gene body of *STAB1* (Fig. 1A), and for each CpG, the OA-associated T allele of rs6976 correlated with lower methylation (Fig. 1B; data for the OA patients only shown). Significant correlations were seen when all of the patient samples were combined (OA knee, OA hip and NOF) as well as in OA samples alone (Table 2).

**Figure 2.**
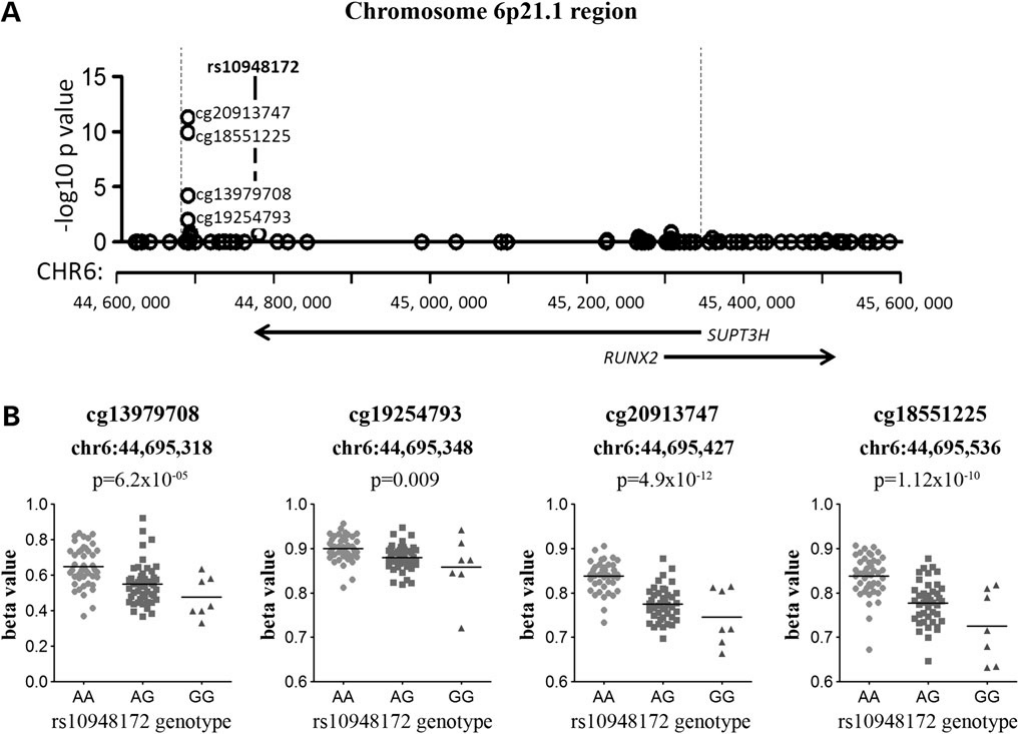
Genotype at rs10948172 correlates with the methylation levels at four CpG sites located downstream of *SUPT3H*. (**A**) The plot shows the association between rs10948172 genotype and the methylation levels of CpG probes that are present within the LD block. The *x*-axis represents the genomic position of the CpG probes, and the *y*-axis represents the Benjamini–Hochberg corrected –log_10_ *P*-value of the correlation between rs10948172 genotype and each CpG probe β-value. Each open circle represents a single CpG probe, the genomic location of rs10948172 is indicated by a bold dashed line, and the LD block is indicated by the vertical dotted lines. The location of the *SUPT3H* and *RUNX2* genes is indicated by arrows. (**B**) The association between genotype at rs10948172 and methylation levels at the four significant CpG probes. Due to the significant methylation differences at these CpGs between OA and NOF samples, data are shown only for the 80 OA samples from the discovery cohort of 99 samples. The level of methylation at the CpG probes is shown as the β-value. Horizontal line represents the mean.

Genotype at the *SUPT3H* SNP rs10948172 correlated with the methylation of four adjacent CpGs located ∼82 kb upstream from the SNP: cg13979708, cg19254793, cg20913747 and cg18551225. All four are located within the LD region marked by rs10948172, which encompasses a ∼667 kb region from hg19 chromosome 6:44 683 049–45 349 877 (Fig. 2A). These four CpG sites are 650 kb downstream of the *SUPT3H* transcription start site (TSS) (Fig. 2A), and for each, the OA-associated G allele of rs10948172 correlated with lower methylation (Fig. 2B; data for the OA patients only shown). rs10948172 is associated with OA in males but not in females (11). However, the meQTL was operating in both males and females (Supplementary Material, Fig. S1), although there is reduced methylation of cg13979708, cg20913747 and cg18551225 in males independent of rs10948172 genotype (Supplementary Material, Fig. S1A, C and D). The meQTL was observed for all of the patient samples combined and in OA samples alone (Table 2).

**Figure 3.**
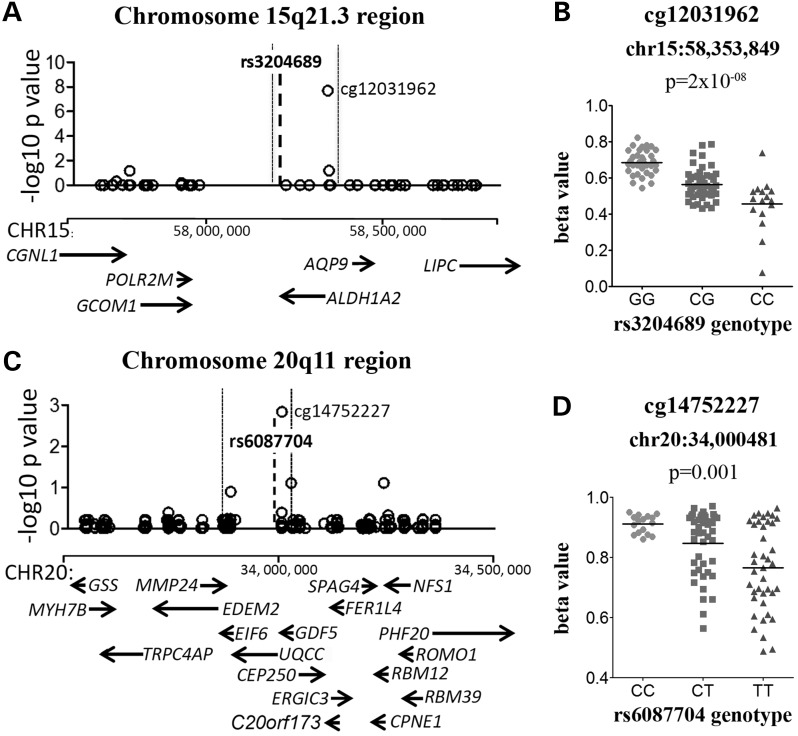
Genotypes at rs3204689 and rs143383 (rs6087704*) correlate with the methylation levels of the cg12031962 and cg14752227 CpG sites, respectively. (**A**). (**C**) Plots show the association between the OA-associated SNP and the methylation levels of CpG probes that are present within each LD block for that SNP. The genomic position of the CpG probes analysed is plotted on the *x*-axis, and the Benjamini–Hochberg corrected –log_10_ *P*-value of the correlation between the genotype and the CpG probe β-value plotted on the *y*-axis. Each open circle represents a single CpG probe, and the significant associations are indicated. The genomic location of the OA-associated SNP is indicated by the bold dashed line, and the LD block is indicated by the vertical dotted lines. The genes within the region analysed are indicated below the association plot by arrows. (**B**), (**D**) Graphs of the association between genotype and methylation β-values for (B) rs3204689-cg12031962 and (D) rs143383-cg14752227. For rs3204689 and rs143383, data are shown for all 99 samples. Horizontal line represents the mean. *rs143383 is not present on the HumanOmniExpress genotyping array and rs6087704, an array SNP that has the highest LD with rs143383 (*r*^2^ = 0.93), was therefore used to infer rs143383 genotypes.

The OA-associated C allele of the *ALDH1A2* SNP rs3204689 correlated with lower methylation at the CpG cg12031962, which is located 107 kb from the SNP (Fig. 3A). This CpG is within the gene body of *ALDH1A2* and within the OA susceptibility region. This meQTL was observed when all of the patient samples were combined (Fig. 3B) and in OA samples only (Table 2).

Genotype at *GDF5* SNP rs143383 correlated with methylation at one CpG, cg14752227, which is located 25 kb from the SNP, 0.5 kb upstream of the TSS of *UQCC* and 25 kb upstream of the TSS of *GDF5* (Fig. 3C). This CpG site resides within the rs143383 LD region and is located 17 bp downstream of the rs62210592 SNP, which has an *r*^2^ of 0.92 with rs143383. The OA-associated T allele of rs143383 correlated with lower methylation of cg14752227 (Fig. 3D; data for all patients shown). This effect was observed for all of the patient samples combined and in OA samples only (Table 2).

### Replication of OA meQTLs

Having identified four OA-associated SNPs correlating with methylation at nine CpG sites, we next attempted to replicate these findings in cartilage DNA samples from additional patients. This replication cohort consisted of 40 OA patients (26 knee and 14 hip) and 5 NOF patients (Supplementary Material, Table S1). As noted above, for rs6976 and rs10948172, significant differences in methylation levels were observed in the discovery study between OA and NOF patients. We therefore excluded the five NOF samples in the replication analysis of these two SNPs. DNA methylation was measured by pyrosequencing. We were unable to design reliable pyrosequencing assays for cg15147215 and cg18591801, both from the chromosome 3p21.1 locus (*GLT8D1*, rs6976), but this left cg18099408 from this locus. In total, therefore, replication assays were designed to cover the remaining seven discovery CpGs. In the design of these assays, seven additional CpGs that were not on the 450k array but which are in close proximity to one or more of the seven discovery CpGs were also captured. These were therefore included in our analysis, such that in total we analysed 14 CpGs, the 7 to be replicated plus an additional 7.

**Figure 4.**
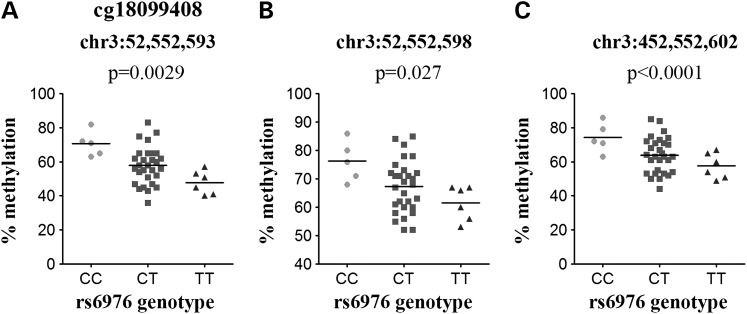
Replication of the association between rs6976 and DNA methylation at cg18099408. (**A**–**C**) Plots show the association between genotype at rs6976 and the methylation levels at three CpGs measured by the pyrosequencing assay; (A) the discovery CpG cg18099408, (B) the CpG site located 5 bp downstream of cg18099408 and (C) the CpG site located 9 bp downstream of cg18099408. Data are shown for the 40 OA samples analysed. *P*-value was calculated using a Kruskal–Wallis test and was Bonferroni-corrected for multiple testing. Horizontal line represents the mean.

The *GLT8D1* rs6976 discovery CpG site cg18099408 replicated in the cohort of 40 OA patients in the same direction as in the discovery cohort (*P* = 0.0029; Fig. 4A). Two additional CpG sites located 5 and 9 bp downstream from cg18099408 were captured by the pyrosequencing assay; these CpG sites also demonstrated significant correlation between rs6976 genotype and methylation, and in the same direction as cg18099408 (Fig. 4B and C).

**Figure 5.**
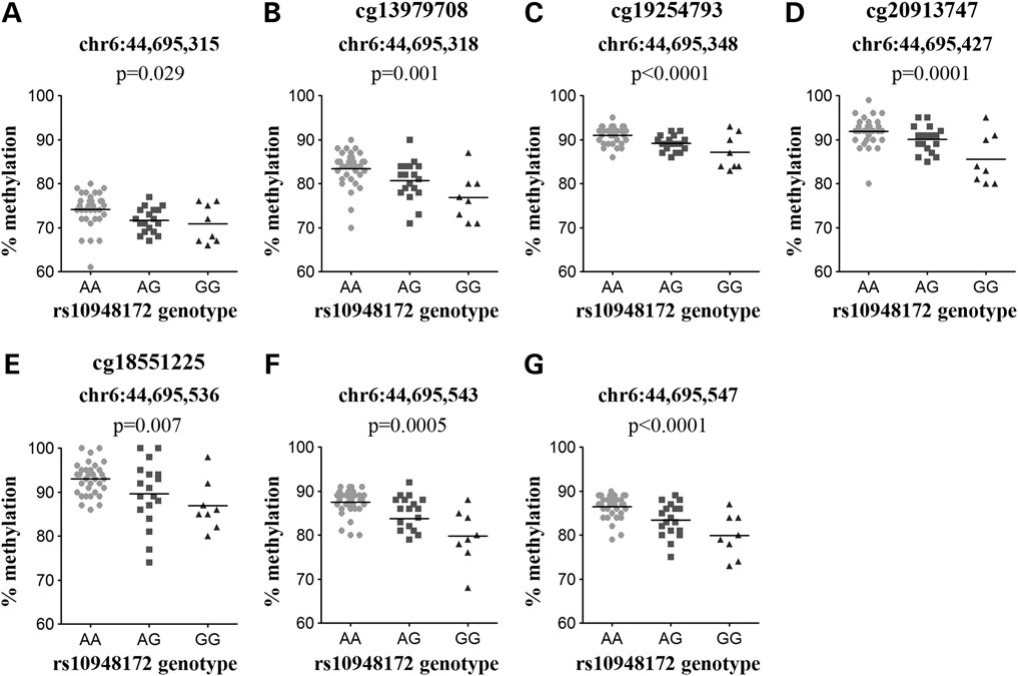
Replication of the association between rs10948172 and DNA methylation. (**A**–**G**) Graphs show the association between genotype at rs10948172 and the methylation levels at seven CpGs measured by pyrosequencing; the four discovery CpGs (B, cg13979708; C, cg19254793; D, cg20913747 and E, cg18551225) and the three additional CpG sites captured by the assays located (A) 3 bp upstream of cg13979708, (F) 7 bp downstream of cg18551225 and (G) 11 bp downstream of cg18551225. Data are shown for the 57 OA samples studied. *P*-value was calculated using a Kruskal–Wallis test and was Bonferroni-corrected for multiple testing. Horizontal line represents the mean.

All four discovery CpGs within the rs10948172 *SUPT3H* meQTL region replicated in the additional cohort, with the OA-associated G allele again correlating with lower methylation (Fig. 5B–E). Additionally, three further CpG sites were captured by the pyrosequencing assays, the first located 3 bp upstream of cg13979708, and the other two located 7 and 11 bp downstream of cg18551225. All three additional CpG sites demonstrated significant correlation between rs10948172 genotype and methylation (Fig. 5A, F and G), and in the same direction as that seen for the four discovery CpGs. This replication analysis of rs10948172 included an additional 17 OA knee patient samples (Supplementary Material, Table S1) that were included to enhance our power to detect any gender differences in methylation independently of genotype at this male-specific susceptibility locus, making a total of 57 OA patient samples (34 females and 23 males). As with the discovery cohort, the effect of rs10948172 genotype on methylation of the seven CpG sites within the meQTL region was observed in both males and females (data not shown), even though the OA genetic risk at this locus is male specific.

**Figure 6.**
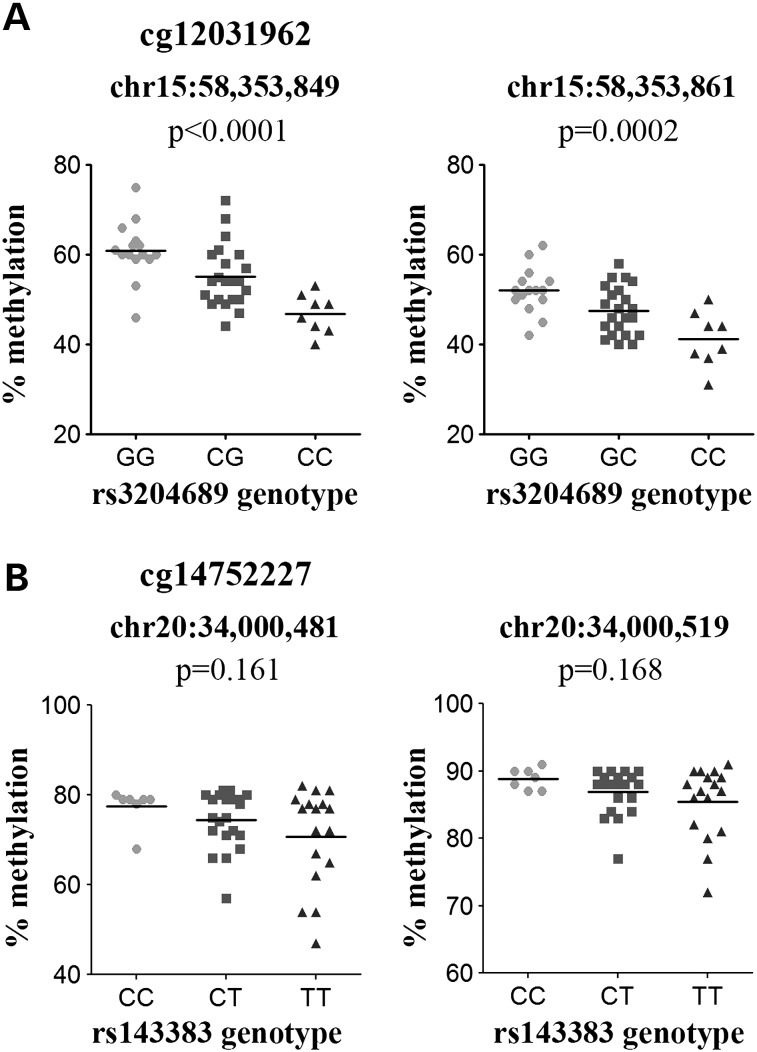
Replication of the (**A**) *ALDH1A2* rs3204689-cg12031962 and (**B**) *UQCC/GDF5* rs143383-cg14752227 meQTLs. For rs3204689, data are shown for the discovery CpG, cg12031962 (left) and an additional CpG located 12 bp downstream (right). For rs143383, data are shown for the discovery CpG, cg14752227 (left) and an additional CpG captured by the assay located 38 bp downstream of cg14752227 (right). For both SNPs, data are shown for the 45 samples studied; 40 OA and 5 NOF. *P*-value was calculated using a Kruskal–Wallis test and was Bonferroni-corrected for multiple testing. Horizontal line represents the mean.

The effect of the *ALDH1A2* rs3204689 SNP genotype on methylation of the cg12031962 CpG was also observed in the additional cohort of 45 cartilage samples (*P* < 0.0001; Fig. 6A, left graph). Additionally, the OA-associated C allele of rs3204689 correlated with reduced methylation of another CpG site captured by the assay that is located 12 bp downstream of cg12031962 (*P* = 0.0002; Fig. 6A, right graph).

The effect of the *GDF5* rs143383 SNP genotype on methylation of cg14752227 was observed in the replication cohort of 45 patients, although this was not significant (Fig. 6B, left graph). This assay captured an additional CpG site located 38 bp away from cg14752227, which also demonstrated a non-significant trend in the same direction as that seen for cg14752227 (Fig. 6B, right graph).

In summary, six of the seven discovery CpGs replicated (*P* < 0.05), and in the same direction as seen previously. Five of the seven additional CpGs captured by the assays also demonstrated a significant correlation between genotype and methylation and in the same direction as their respective discovery CpGs. Three of the four OA meQTLs therefore replicated, while the *GDF5* meQTL demonstrated a non-significant trend towards replication.

### The impact of OA meQTLs on gene expression

We next assessed whether the meQTLs correlated with gene expression. For 29 of the 63 OA knee patients used in the discovery phase of our meQTL analysis, we had RNA available from the same cartilage samples. For the four meQTL loci, we assayed the expression of 11 genes that are known to be expressed in cartilage (4,5,11,13,37). For each gene, we correlated the expression level with the methylation level for each discovery CpG from that mQTL locus. For example, for the rs6976 locus, a correlation between expression for each of the six cartilage-expressed genes and the level of methylation at each of the three discovery CpGs (cg18099408, cg15147215 and cg18591801) was made.

**Figure 7.**
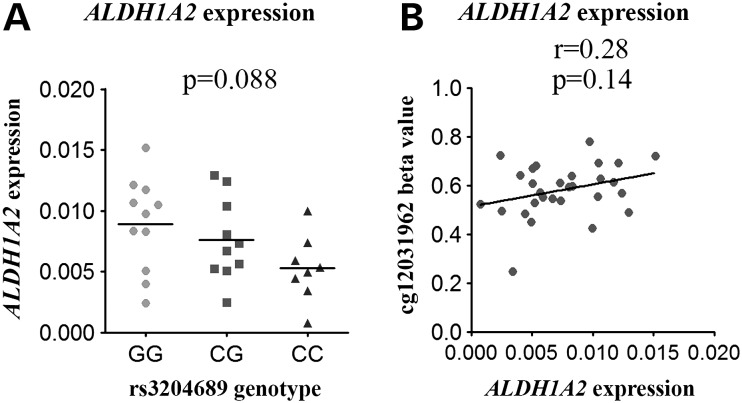
eQTL and methylation–expression correlations for *ALDH1A2*. (**A**) *ALDH1A2* expression in cartilage from 29 OA knee patients stratified by genotype at rs3204689. Horizontal line represents the mean. (**B**) *ALDH1A2* expression plotted against methylation at cg12031962. *r* represents the Spearman rank coefficient and the *P*-value was calculated using a Kruskal–Wallis test and was Bonferroni-corrected for multiple testing.

None of the genes showed a significant correlation (*P* < 0.05; Spearman correlation) between gene expression and methylation, or between expression and genotype (Fig. 7; Supplementary Material, Figs S2 and S3). We did, however, observe a non-significant trend between *ALDH1A2* expression and genotype at rs3204689 (*P* = 0.088; Fig. 7A), with the risk-conferring C allele correlating with reduced expression; this fits with the previous eQTL analysis of this locus in OA cartilage (13). There was also a non-significant positive correlation between *ALDH1A2* expression and cg12031962 methylation in OA knee cartilage (Fig. 7B).

### Analysis of OA meQTLs in other cell and tissue types

We next analysed published studies that used the 450k array to determine whether any of the cartilage meQTLs that we had identified were also active in other cell or tissues types. This revealed that each of our cartilage meQTLs operate as meQTLs in non-joint tissues, with the *SUPT3H*/*RUNX2* cg20913747 and the *ALDH1A2* cg12031962 signals demonstrating the most widespread effects, with meQTL activity in at least six cell/tissue types (Table 3). To assess whether these two meQTLs also operate in non-cartilaginous joint tissues from OA patients, we examined methylation at these sites in DNA extracted from the synovium and infrapatellar fat pad of OA knee patients. We also analysed whole-blood DNA from OA knee patients.

**Table 3.** Cartilage meQTLs active in non-joint tissues

Chromosome 3p21.1	Chromosome 6p21.1			Chromosome 15q21.3	Chromosome 20q11
*GNL3/GLT8D1*	*SUPT3H/RUNX2*			*ALDH1A2*	*UQCC/GDF5*
rs6976	rs10948172			rs3204689	rs143383
cg15147215	cg19254793	cg20913747	cg18551225	cg12031962	cg14752227
Pancretic islets (33; *r*^2^ = 0.72)	Pancreatic islets (33)	Pancreatic islets (33)	Pancreatic islets (33)		
		Lymphocytes (38; *r*^2^ = 0.96)		Lymphocytes (38)	Lymphocytes (38; *r*^2^ = 0.89)
	Adipose (32; *r*^2^ = 0.78)	Adipose (32; *r*^2^ = 0.96)		Adipose (32)	Adipose (32; *r*^2^ = 0.92)
		Whole blood (32; *r*^2^ = 0.96)		Whole blood (32)	Whole blood (32; *r*^2^ = 0.92)
	Fibroblasts (39; *r*^2^ = 0.96)	Fibroblasts (39; *r*^2^ = 0.96)		Fibroblasts (39; *r*^2^ = 0.94)	
		T lymphocytes (39,40; *r*^2^ = 0.96)		T lymphocytes (39,40; *r*^2^ = 0.94)	
				Lymphoblastoid (39; *r*^2^ = 0.94)	
				Whole blood (31)	
		Lung (41; *r*^2^ = 0.92)	Lung (41; *r*^2^ = 0.92)		

Whole blood from 247 donors (31). Adipose tissue from 648 donors and peripheral blood from 200 donors (32). Pancreatic islets from 89 donors (33). Lymphocytes from 989 donors with colon cancer and 850 controls (38). Umbilical cord fibroblasts from 107 donors, T-cells from 66 donors and transformed lymphoblastoid cells from 111 donors (39,40). Lung tissue from 210 donors (41). Published meQTL data sets generated using the 450k array were interrogated for the four cartilage meQTLs identified in this study (references are indicated in brackets). If the SNP used in a published report is not the same as that used by us (or is a different SNP, which is not in perfect LD with the OA SNP), then its *r*^2^ value relative to the OA SNP is shown.

**Figure 8.**
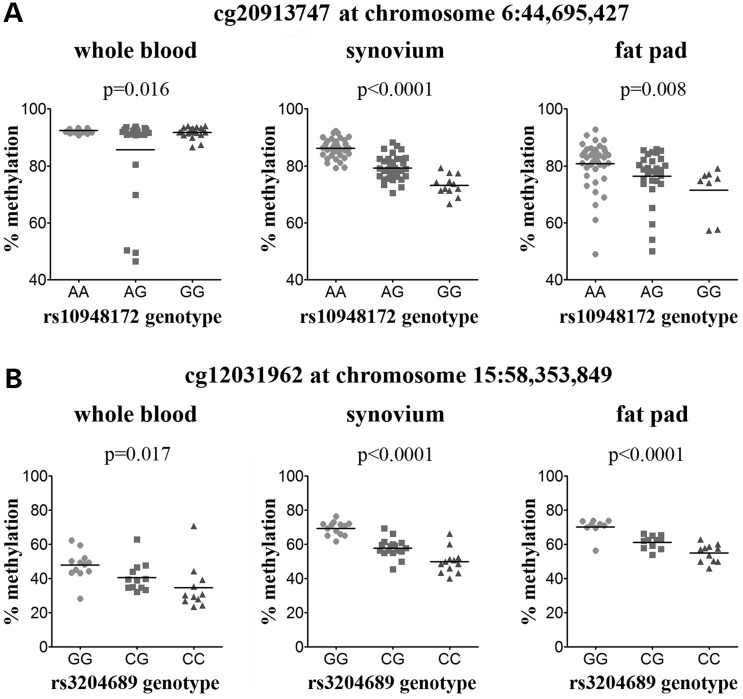
meQTL analysis of rs10948172-cg20913747 and rs3204689-cg12031962 in whole blood, synovium and fat pad from OA knee patients. (**A**) Methylation at cg20913747 stratified by genotype at the *SUPT3H/RUNX2* OA SNP rs10948172. (**B**) Methylation at cg12031962 stratified by genotype at the *ALDH1A2* OA SNP rs3204689. The *P*-value was calculated using a Kruskal–Wallis test and was Bonferroni-corrected for multiple testing. Horizontal line represents the mean.

For the *SUPT3H*/*RUNX2* rs10948172 meQTL, we investigated the CpG cg20913747, which is subject to an rs10948172 meQTL in seven tissues in the published reports (Table 3). Furthermore, this CpG site showed the most significant effects in our cartilage data (Figs 2B and 5D), with genotype accounting for 27.8 and 24.6% of the total inter-individual variability in cg20913747 methylation in the discovery and replication cohorts, respectively. Although overall methylation was lower in synovium and fat pad than in cartilage, we observed a significant effect of rs10948172 genotype on cg20913747 methylation in all three tissue types in the same direction as in cartilage (Fig. 8A); rs10948172 genotype accounted for 63.1, 14.1 and 6.7% of cg20913747 methylation variability in synovium, fat pad and blood, respectively. For synovium and fat pad, this meQTL was independent of gender (Supplementary Material, Fig. S4B), although we observed a reduction in fat pad methylation in males relative to females that was independent of genotype (*P* = 0.009; Supplementary Material, Fig. S4). There is a non-significant decrease in methylation of cg20913747 in whole blood from males (Supplementary Material, Fig. S4A), and the effect of rs10948172 genotype on methylation appears to be male specific in this tissue (Supplementary Material, Fig. S4B). This, combined with the small effect size observed in blood relative to synovium and fat pad, may explain why this meQTL was not identified in other studies of whole blood (31,32).

Analysis of whole blood, synovium and fat pad DNA revealed that methylation levels of the *ALDH1A2* CpG site cg12031962 vary between tissues, with the lowest methylation observed in whole blood (mean of 41.2%, *n* = 33) and the highest methylation observed in fat pad (mean of 61.6%, *n* = 31). Despite these differences in methylation levels, the rs3204689-cg12031962 meQTL is present in all three tissues (Fig. 8B) and in the same direction as seen in cartilage, with the OA-associated C allele of rs3204689 being associated with lower methylation relative to the G allele.

## Discussion

We aimed to investigate whether OA-associated loci impact upon DNA methylation in cartilage in *cis* and are therefore functioning as meQTLs. Four of the 16 loci that we tested demonstrated meQTL activity in the discovery phase, and three demonstrated statistically significant replication. As far as we are aware, this is the first study to identify OA association signals operating as meQTLs. Going forward, the identification and analysis of meQTLs will be a powerful tool in interpreting the functional effect of OA risk alleles. Three replicated meQTLs from a total of 16 loci analysed represent a 18.7% frequency of OA risk loci acting as meQTLs. This figure is comparable to the 10.7% of type II diabetes loci that also act as meQTLs (33).

We investigated methylation changes that occur within and immediately adjacent to the LD block of each association signal, and therefore, our search was focussed on *cis*-meQTLs. While there is compelling evidence for the existence of *trans*-acting meQTLs operating in common polygenic diseases, to date it has been observed that the overwhelming majority of meQTLs operate in *cis*. These *cis* affects can act over a range of distances, from a few base pairs to distances of over 500 kb, with the affected CpG site being outside of the meQTL SNP LD region (40,41). This is the case for the *GLT8D1* rs6976 meQTL, where the three CpGs affected by genotype at this SNP are located ∼31.5 kb upstream of the rs6976 LD region. This suggests physical interaction between the association interval and CpGs nearby but outside of the LD region. Further to this, there is evidence that proximal CpG sites may mediate the effect of *trans*-meQTLs on more distal CpG sites (41). Although prioritising *cis*-meQTLs is therefore justified, we cannot rule out the presence of *trans-*acting meQTLs operating on OA association signals. However, *trans*-meQTLs have been shown to typically have a smaller effect size than *cis*-meQTLs, and their discovery would therefore likely require larger sample sizes than those in our study.

Although we observed that 4 of the 16 loci are functioning as *cis*-meQTLs, we are unable to discount the possibility that the remaining 12 also act through the *cis*-regulation of DNA methylation. For example, several of the OA associations are hip specific, and it may be that their effects are only active in hip cartilage and that our relatively low number of such samples lacked power. Furthermore, the methylation array that we used captures <5% of the CpGs in the human genome and as such it could miss effects at other CpGs. Our study therefore provides impetus to both analyse an even larger data set and to expand the number of CpGs investigated. For example, our previous analysis of the 5′ untranslated region (UTR) of *GDF5* identified several CpG sites whose methylation was altered in OA cartilage (35). However, none of these CpGs are assayed by the 450k array used in this current study.

All four of the cartilage meQTLs that we discovered have been previously reported to be operating as meQTLs in other tissues. This observation is consistent with a previous study that showed that while DNA methylation patterns are highly tissue specific, meQTLs can operate across numerous tissues as well as during different development stages (42). We directly tested two of our meQTLs, the *SUPT3H*/*RUNX2* rs10948172-cg20913747 meQTL and the *ALDH1A2* rs3204689-cg12031962 meQTL, in other joint tissues and whole blood from OA knee patients. Even though methylation levels at these two CpG sites varied between cartilage, blood, synovium and fat pad, the effect of genotype on methylation was observed in all four tissues. However, for the rs10948172-cg20913747 meQTL, gender also had an effect on methylation in cartilage, blood and fat pad. In blood, the meQTL was only present in males, with no effect of rs10948172 genotype observed in females. In contrast, the meQTL was observed in both genders in cartilage and fat pad, although there is reduced methylation of cg20913747 in males relative to females independently of rs10948172 genotype in both tissues. The effect of gender on cg20913747 is of note given that the OA risk conferred by the *SUPT3H*/*RUNX2* SNP rs10948172 is male specific, and that the OA risk G allele is associated with reduced methylation of cg20913747.

In our study, we stratified methylation by genotype only at the OA-associated SNPs identified in previous genetic studies; although these SNPs themselves may not be the causal OA risk SNP, they act as a marker of the OA susceptibility LD block. We did not stratify the methylation data by genotype of other SNPs within the analysed regions. We cannot therefore rule out the possibility that additional SNPs, either those in high LD or not in LD with the OA SNPs, act as meQTLs on the identified CpG sites, and that these SNPs may actually drive the meQTLs that we detected. However, the published meQTL studies of other tissues that we analysed used a genome-wide approach to identify meQTLs, and in these studies, only the most significant meQTL for each CpG site was reported. For the cartilage meQTLs identified in our study that were present in these other tissues, the SNPs that had the most significant effect on methylation in these tissues had an *r*^2^ of between 0.72 and 1 with the OA-associated SNP used in our study. For the meQTLs that are present in the largest number of tissues, the *SUPT3H*/*RUNX2* rs10948172-cg20913747 and the *ALDH1A2* rs3204689-cg12031962 meQTLs, the most significant SNPs identified in the genome-wide meQTL studies had an *r*^2^ of between 0.92 and 1 with the OA SNP. Assuming that the SNP responsible for the meQTL is the same in all tissues, this suggests that the four cartilage meQTLs we identified are driven by SNPs in high or perfect LD with the OA-associated SNPs that we used in our study rather than being driven by other SNPs within the analysed regions that are not in LD with the OA susceptibility SNPs.

The effect of methylation on gene expression is well described, and it is therefore reasonable to expect that genetic variants that impact on DNA methylation would also impact on gene expression. For example, 6% of adipose meQTLs are also associated with gene expression in adipose tissue (32), while causal inference testing (CIT) has identified 10 CpG sites that mediate genetic risk for RA through effects on gene expression (31). However, none of the four meQTLs that we identified had a significant effect on gene expression in cartilage. This could be because they are not functional, or because any expression effects are too small for us to have detected with our sample size of 29 patients. It is well known that regulatory regions are functional only during specific points of cellular differentiation and tissue formation (43). It may therefore be that the methylation signatures for the meQTLs that we detected are enduring markers of an effect on gene expression that occurred earlier in the development of the cartilage tissue. For example, they may have influenced the expression of nearby genes, which led to altered protein levels, which impacted detrimentally on tissue integrity but which only manifested as a pathology as the individual aged. We did observe that genotype at rs3204689 showed a non-significant trend with gene expression of *ALDH1A2*. The association of rs3204689 is with hand but not with hip or knee OA (13). However, the group that reported that association signal used hip and knee cartilage to assess the functional impact of the association signal on *ALDH1A2* expression. This was presumably because of the lack of cartilage tissue available from the finger joints of OA patients, which are not typically operated upon. They discovered that the risk-conferring C allele of rs3204689 is expressed at a lower level than the G allele. Our *ALDH1A2* expression data support this finding and reiterate that for functional analyses, one can use cartilage from a skeletal site that does not genetically associate with OA.

Although DNA methylation has historically been studied in the context of gene repression, recent studies have identified other potential roles of DNA methylation in regulating intergenic/cryptic start sites, splicing and as a biomarker of histone modifications and transcription factor binding (reviewed in Ref. 44). DNA methylation levels can act as a marker to distinguish exons from introns, with methylation being more abundant in exons relative to the flanking introns, and with methylation levels being higher in constitutively spliced exons than alternatively spliced exons (reviewed in Ref. 45). Several gene-specific and genome-wide studies in different species have connected DNA methylation to the regulation of alternative splicing, one example being the demethylation-associated changes in alternative splicing observed in honey bees after inhibition of the *Dnmt3* DNA methyltransferase (46). DNA methylation has also been implicated in the regulation of alternative promoters, with tissue-specific DNA methylation of two intragenic promoters within the human *SHANK3* gene inversely correlating with transcription from these alterative promoters *in vitro* and *in vivo* (47). Additionally, in the brain, unmethylated intragenic CpG islands overlap with the histone modification H3K4me, a marker of active promoters, suggesting that they may function as alternative promoters (47). Both the rs6976-cg15147215 and rs3204689-cg12031962 meQTLs are located intragenically and so may affect alternative splicing or transcription from alternative promoters, neither of which would have been detected by our gene expression assays. Future RNA-seq-based expression analyses of cartilage would be able to address these possibilities.

For all four meQTLs, we observed the methylation effect when only OA cartilage samples were analysed and when the OA and the non-OA NOF samples were combined. This suggests that the effect of genotype on methylation is not disease driven. This fits with the polygenic model of common disease aetiology and confirms the validity of using non-diseased tissues when assessing the effect of a risk-conferring locus (48).

In our study, we specifically focussed on the relationship between DNA methylation and SNPs that have been reported to be associated with OA at a high degree of statistical significance. In a previous report on the DNA methylation of OA cartilage, den Hollander and colleagues (49) correlated global gene expression with DNA methylation between cartilages located at and away from the OA lesion. They identified 70 genes that showed both expression and methylation differences between the two types of cartilage, and for 23 of these 70 genes, proximal SNPs, located within 10 kb of the genes, were found to correlate with expression/methylation levels. None of these SNPs are within the OA association signals studied by us. This study, therefore, highlights an alternative means of identifying meQTLs that could impact on OA pathology.

In conclusion, we have identified the first meQTLs that correlate with OA genetic association signals. These were detected in cartilage, confirming the important role of this tissue in the disease process, while two of them were also observed in other joint tissues from OA patients, emphasizing the fact that effects on non-cartilaginous tissue also need to be considered when studying the molecular pathology of OA (50). Furthermore, the meQTLs are active in non-joint tissues and as such are not OA specific. The identification of OA meQTLs greatly assists our interpretation of OA susceptibility by demonstrating a functional link between genetics and epigenetics for this common, age-related disease.

## Materials and Methods

### Nucleic acid extraction from patient tissues

Patients were ascertained and their tissues collected as described in our previous publications (5,16,24). The Newcastle and North Tyneside research ethics committee granted ethical approval for the tissue collection (REC reference number 09/H0906/72), and informed consent was obtained from each donor. Genomic DNA was isolated from ground cartilage, synovium or infrapatellar fat pad using an EZNA DNA/RNA isolation kit (Omega Bio-Tek), as previously described (24). DNA was extracted from peripheral whole blood using the QIAamp DNA blood mini kit (Qiagen). For genotyping, the DNA was used directly, whereas for methylation analysis, the DNA was bisulphite converted using the EZ DNA methylation kit (Zymo Research). RNA was isolated from the ground cartilage using the RNeasy kit (Qiagen), treated with Turbo DNase (Ambion) and then reverse transcribed using the SuperScript First-Strand cDNA synthesis kit (Invitrogen).

### Genotyping the 16 SNPs in the discovery data set

We investigated 16 SNPs that have previously been shown to be associated with OA (Table 1; 4–14). Genotypes were determined using a genotyping array, a pyrosequencing assay or restriction fragment length polymorphism (RFLP) analysis (Supplementary Material, Table S2).

We investigated 99 patients in our discovery of the OA meQTLs. For 90 of these patients, genotypes at the 16 SNPs were determined using data from the HumanOmniExpress Bead Chip array (Illumina). Standard quality control (QC) procedures were carried out in PLINK version 1.07 with visualization performed in R (http://www.r-project.org/, accessed 26 October 2015) (51). For the remaining nine discovery patients, genotypes at the 16 SNPs were determined using pyrosequencing or RFLP assays. Supplementary Material, Table S2 provides details of the assays, including the sequences of the primers used. An independent cohort of cartilage from 62 patients were examined to replicate the four meQTLs. Genotypes for the four relevant SNPs (rs6976, rs10948172, rs3204689 and rs143383) were determined in these patients using pyrosequencing or RFLP assays.

### Discovery of meQTLs

Processed and normalized cartilage DNA methylation data were acquired from our previous study (24), in which we had used Illumina's Infinium HumanMethylation450 BeadChip. For each CpG probe, the data from the array are represented as a β-value that ranges from 0 (no methylation) to 1 (100% methylation). CpGs within the entire LD block (defined as *r*^2^ > 0.8 with the association SNP) of each of the 16 SNPs were tested. For 15 of the OA loci, 1 Mb encompassed the LD block while for rs10948172, the region of analysis was increased to 1.5 Mb to cover its LD block. Polymorphic CpG sites (i.e. those created by a SNP) were excluded from our analysis. Linear regression was used to measure the relationship between the β-value and the genotype at the OA association SNP. β-Values were modelled as a linear function of the number of minor alleles (0, 1 or 2), as has been previously reported (42,52). *P-*values were corrected for multiple testing using the Benjamini–Hochberg method and were adjusted for the total number of CpG probes that were within the tested region surrounding the association SNP (Table 1). For example, the 1 Mb region surrounding rs11842874 (Locus 9) contained 655 CpG probes and so the correction was made for 655 tests. Genotype–methylation correlations were deemed to be significant at *P* < 0.05 after Benjamini–Hochberg correction.

### Replication of meQTLs in cartilage, whole blood, synovium and fat pad

CpG methylation analysis of the replication cohort was performed by pyrosequencing. The assays were designed using PyroMark assay design SW 2.0 (Qiagen), and sequencing was performed using PyroMark Q24 (Qiagen) as previously described (34). The pyrosequencing primer sequences are listed in Supplementary Material, Table S3. For each sample, PCR reactions were formed in duplicate and the mean calculated, with samples being excluded from the analysis if the methylation between the PCR replicates differed by >5%. For each CpG site analysed, a Kruskal–Wallis test with Bonferroni correction for multiple testing was performed using GraphPad Prism software, and genotype–methylation correlations were deemed significant if the adjusted *P-*value was <0.05. Percentage methylation variability accounted for by genotype was calculated using a general univariate linear model with the SPSS Statistics software.

### Gene expression

Relative gene expression was measured by real-time PCR using TaqMan^®^ chemistry. Predesigned TaqMan^®^ assays (Integrated DNA technologies) covering all known transcript variants were used. Expression of target genes was measured relative to the housekeeping genes *18S*, *GAPDH* and *HPRT1*. The relative expression of the target genes was calculated using the 2^−ΔCt^ method: 2^−ΔCt^ (target gene) = 2 ^–[Ct (target gene) – Ct (average of housekeeping genes^^)]^. Outliers were removed using the Grubb's test, and a *t*-test was used to assess the differences in gene expression.

## Supplementary Material

Supplementary material is available at *HMG* online.

## Funding

This work was supported by Arthritis Research UK, by the NIHR Newcastle Biomedical Research Centre, by the Medical Research Council and Arthritis Research UK as part of the MRC-Arthritis Research UK Centre for Integrated Research into Musculoskeletal Ageing (CIMA) and by the European Union's Seventh Framework Program for research, technological development and demonstration under grant agreement number No. 305815 (D-BOARD). Funding to pay the Open Access publication charges for this article was provided by the European Union's Seventh Framework Program for research, technological development and demonstration under grant agreement number No. 305815 (D-BOARD).

## Supplementary Material

Supplementary Data
