# Supplementary material for: Methylation quantitative trait locus analysis of osteoarthritis links epigenetics with genetic risk
Source: Hum Mol Genet. 2015 Oct 13;24(25):7432–44. doi: 10.1093/hmg/ddv433 (PMC4664171; doi:10.1093/hmg/ddv433)
Supplement: Supplementary Data [file supp_24_25_7432__index.html]

Methylation quantitative trait locus (meQTL) analysis of osteoarthritis links epigenetics with genetic risk — Methylation quantitative trait locus analysis of osteoarthritis links epigenetics with genetic risk — Methylation quantitative trait locus analysis of osteoarthritis links epigenetics with genetic risk — Supplementary Data 

# Methylation quantitative trait locus analysis of osteoarthritis links epigenetics with genetic risk

## Supplementary Data

Supplementary Data

- Supplementary Data - Docx file
- Supplementary Table 1 - xlsx file
- Supplementary Table 2 - xlsx file
- Supplementary Table 3 - docx file
