# Supplementary material for: Methylation quantitative trait locus analysis of osteoarthritis links epigenetics with genetic risk
Source: Hum Mol Genet. 2015 Oct 13;24(25):7432–44. doi: 10.1093/hmg/ddv433 (PMC4664171; doi:10.1093/hmg/ddv433)
Supplement: Supplementary Data [file supp_ddv433_ddv433supp.docx]

**
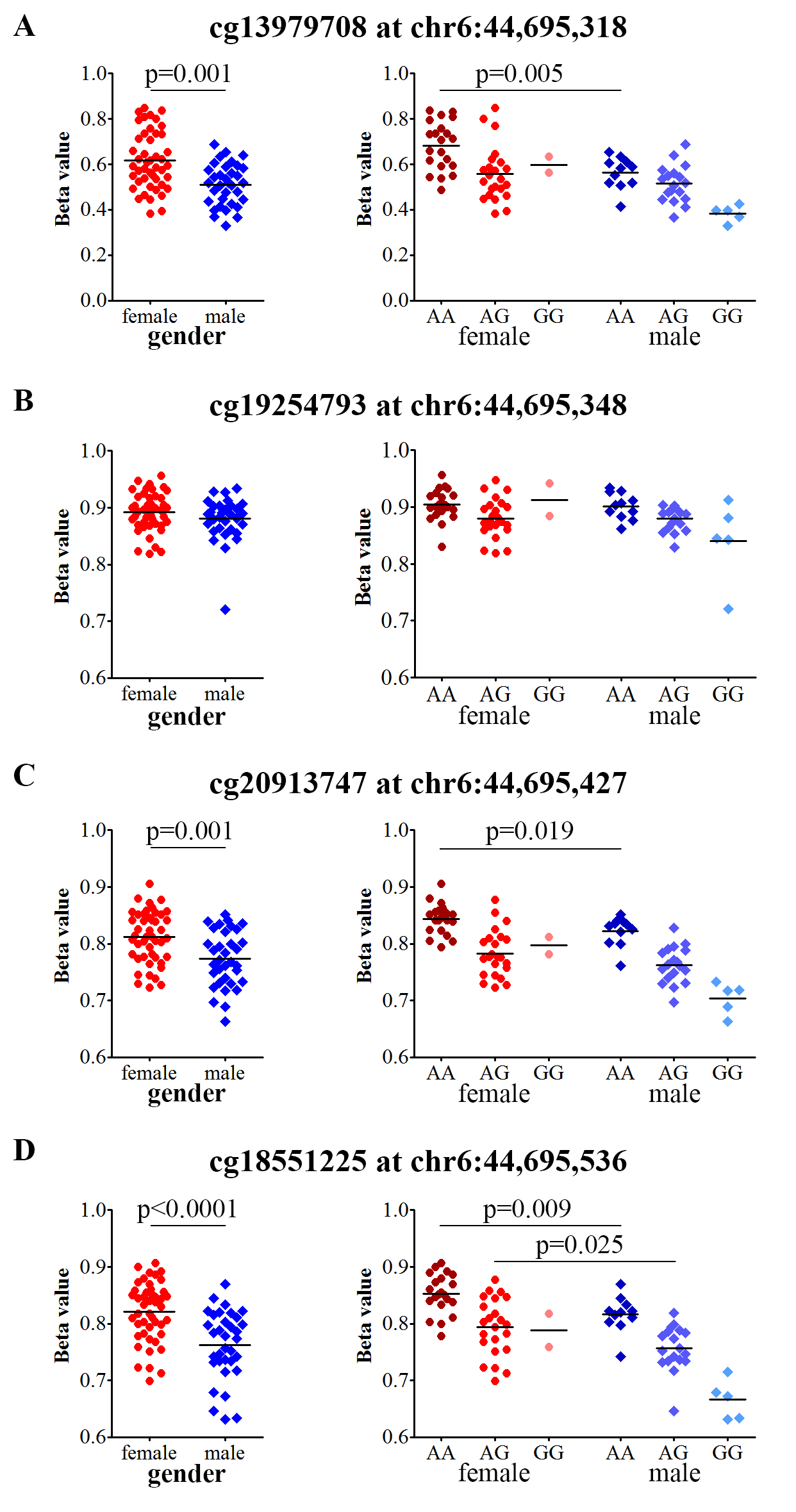
**

**Supplementary Material, Figure S1.** Gender stratification of the *SUPT3H/RUNX2* rs10948172 meQTL discovery cohort. Beta values for **(A)** cg13979708, **(B)** cg19254793, **(C)** cg20913747 and **(D)** cg18551225 were stratified by gender alone (left side) or gender and genotype (right side). For gender only analyses, the p value was calculated using a Mann-Whitney U test. For combined gender-genotype analyses, methylation levels in females and males of the same genotype were compared and the p value calculated using a Kruskal-Wallis test with Bonferroni multiple testing corrections. A minimum of three values are needed per condition and as there were only two female GG samples, it was not possible to statistically compare methylation between male and female GG homozygotes.

**
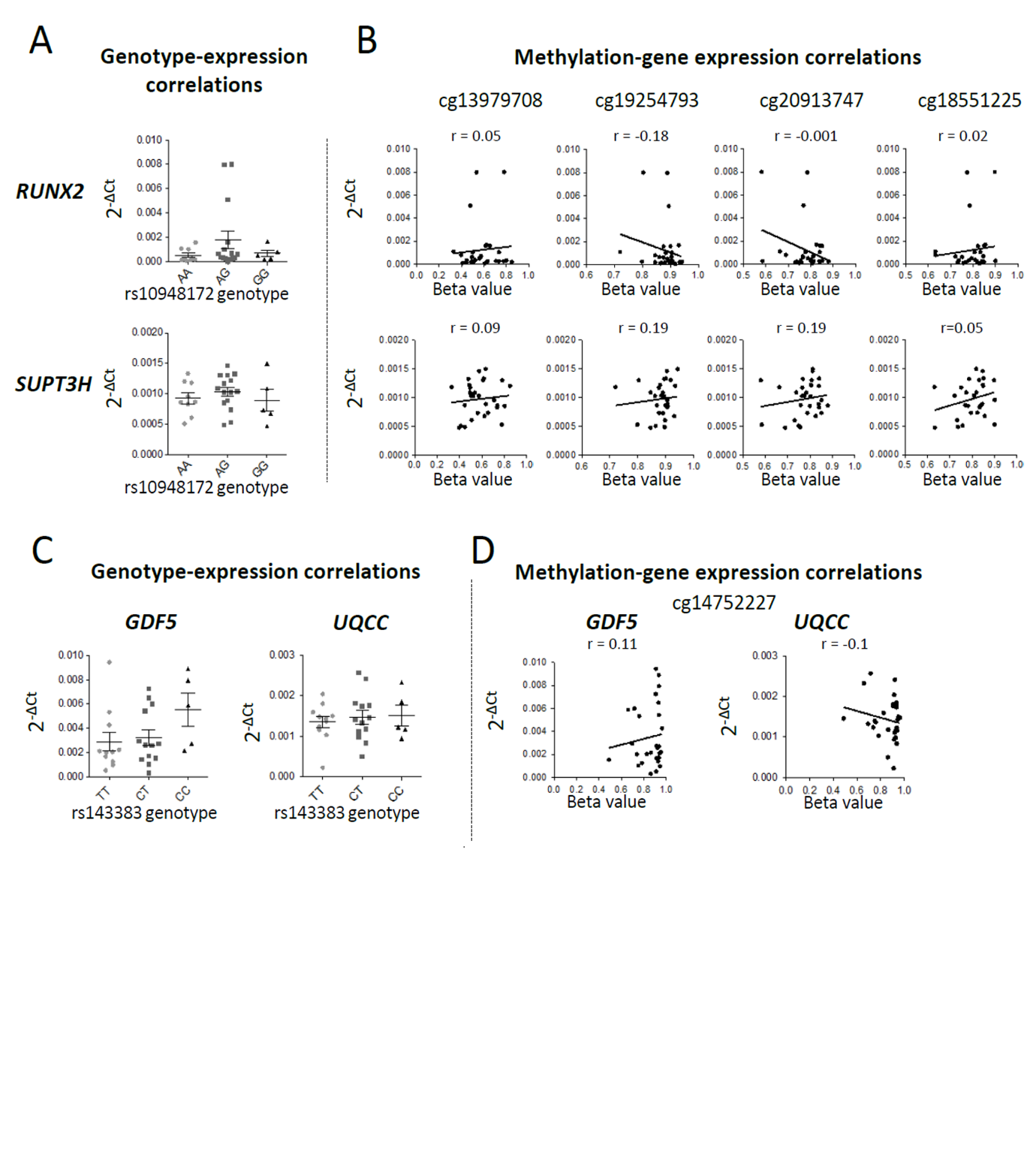
**

**Supplementary Material, Figure S2.** Correlation between gene expression and methylation at rs10948172 and rs143383. (**A**) Plots show the correlation between genotype at rs10948172 (x-axis) and the expression of *RUNX2* and *SUPT3H* (y-axis). (**B**) Plots show the correlation between gene expression of *RUNX2* and *SUPT3H* (y-axis) and methylation (x-axis) at the four CpGs that correlate with genotype at rs10941872. (**C**) Plots show the correlation between genotype at rs143383 (x-axis) and the expression of *GDF5* and *UQCC* (y-axis). (**D**) Plots show the correlation between gene expression of *GDF5* and *UQCC* (y-axis) and methylation (x-axis) at the CpG site that correlates with genotype at rs143383. r represents the Spearman rank coefficient. None of the correlations were significant using a p value cut off of < 0.05.





**Supplementary Material, Figure S3.** Correlation between gene expression and methylation at rs6976. (**A**) Plots show the correlation between genotype at rs6976 (x-axis) and the expression of the six genes that are within the rs6976 locus (y-axis). (**B**) Plots show the correlation between gene expression (y-axis) and methylation at the three CpGs that correlate with genotype at rs6976 (x-axis). r represents the Spearman rank coefficient. None of the correlations were significant using a p value cut off of < 0.05.


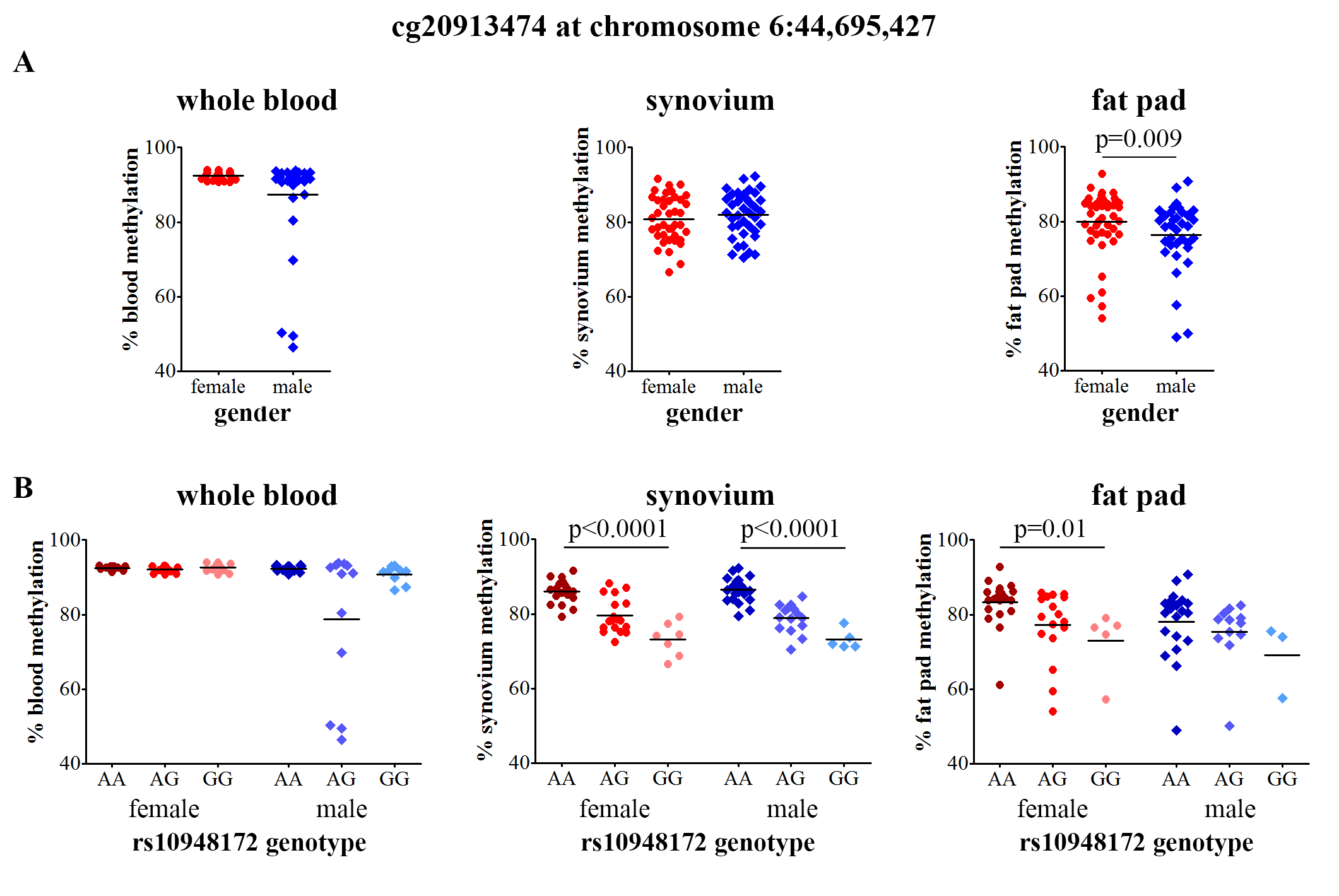


**Supplementary Material, Figure S4.** Gender stratification of the *SUPT3H/RUNX2* rs10948172-cg20913474 meQTL in whole blood, synovium and fat pad from OA knee patients. Methylation was measured by bisulphite pyrosequencing and stratified by **(A)** gender and **(B)** gender and genotype at rs10948172. For gender only analyses, the p value was calculated using a Mann-Whitney U test. For combined gender-genotype analyses, the p value for genotype stratification for each gender was calculated using a Kruskal-Wallis test and Bonferroni corrected for multiple testing.
