# Supplementary material for: Methylation quantitative trait locus analysis of osteoarthritis links epigenetics with genetic risk
Source: Hum Mol Genet. 2015 Oct 13;24(25):7432–44. doi: 10.1093/hmg/ddv433 (PMC4664171; doi:10.1093/hmg/ddv433)
Supplement: Supplementary Data [file supp_ddv433_ddv433supp_table3.docx]

**Supplementary Table 3.** Primer sequences of the pyrosequencing assays used in the methylation analysis of CpGs for the replication study. Probe represents the CpG from the Illumina 450k methylation array that the assay measures. Btn = biotinylated, indicating the biotin-labeled primer.

| **Region** | **Probe** | **Forward primer** | **Reverse primer** | **Sequencing primer** |
| --- | --- | --- | --- | --- |
| ***GNL3*/*GLT8D1*** | **cg18099408** | Btn 5 ´GTTAGGGGGTTGGTTGGTTT 3´ | 5´ CCCAACCTCRCTCTAAAAACT 3´ | 5´ CAAATTACTCATTAAATCTA 3´ |
|  | **cg15147215** | 5´ GGATGAGTTGGTTYGGATT 3´ | Btn 5´ AAAACRCCCCCTAACTAAAAA 3´ | 5´ GGATTTGTTGGAGTAGGGGT 3´ |
|  | **cg18591801** | Btn 5´ GTTGTTTTTCGAGGCGTTGT 3´ | 5´ CCAAACCCCCACCTACCTAT 3´ | 5´ TATCTTCCAACTCCCAAAC 3´ |
| ***SUPT3H/RUNX2*** | **cg13979708** | Btn 5´ AGGTTGGTAATAGTAGTAGTTAGGTTAGTG 3´ | 5´ AACAAATACCAACCCTCCCAAAATTCT 3´ | 5´ AAAATTATCCAAATCTTCCT 3´ |
|  | **&** |  |  |  |
|  | **cg19254793** |  |  |  |
|  | **cg20913747** | 5´ AGGTTGGTAATAGTAGTAGTTAGGTTAGTG 3´ | Btn 5´ AACAAATACCAACCCTCCCAAAATTCTAC 3´ | 5´ ATAAGATAGGAGTTGAGTATTATAA 3´ |
|  | **cg18551225** | 5´ TTTTGGGAGGGTTAGTATTTGTTAAGTATG 3´ | Btn 5´ CCTACTTCTTACCAACTCCTTCTAACTACC 3´ | 5´ TTGAAGTTAGAAAGTGAG 3´ |
| ***ALDH1A2*** | **cg12031962** | Btn 5´ TTTTGGTATAGGTAAGAATTTTGTTTT 3´ | 5´ CATCACCATACACTTTTTCTAAC 3´ | 5´ ATTTTCATCACCATACACTT 3´ |
| ***UQCC/GDF5*** | **cg14752227** | Btn 5´ GTYGGGTATGTTGGTGTGTG 3´ | 5´ CCAACCACAAATCCCAAAAAT 3´ | 5´ ACCAAACTTCAAATCAACAC 3´ |
|  |  |  |  |  |
